# Supplementary material for: Anti-HER2 Cancer-Specific mAb, H2Mab-250-hG1, Possesses Higher Complement-Dependent Cytotoxicity than Trastuzumab
Source: Int J Mol Sci. 2024 Aug 1;25(15):8386. doi: 10.3390/ijms25158386 (PMC11313270; doi:10.3390/ijms25158386)
Supplement: Supplementary file 1 [file ijms-25-08386-s001.zip › ijms-2999806-supplementary.pdf]

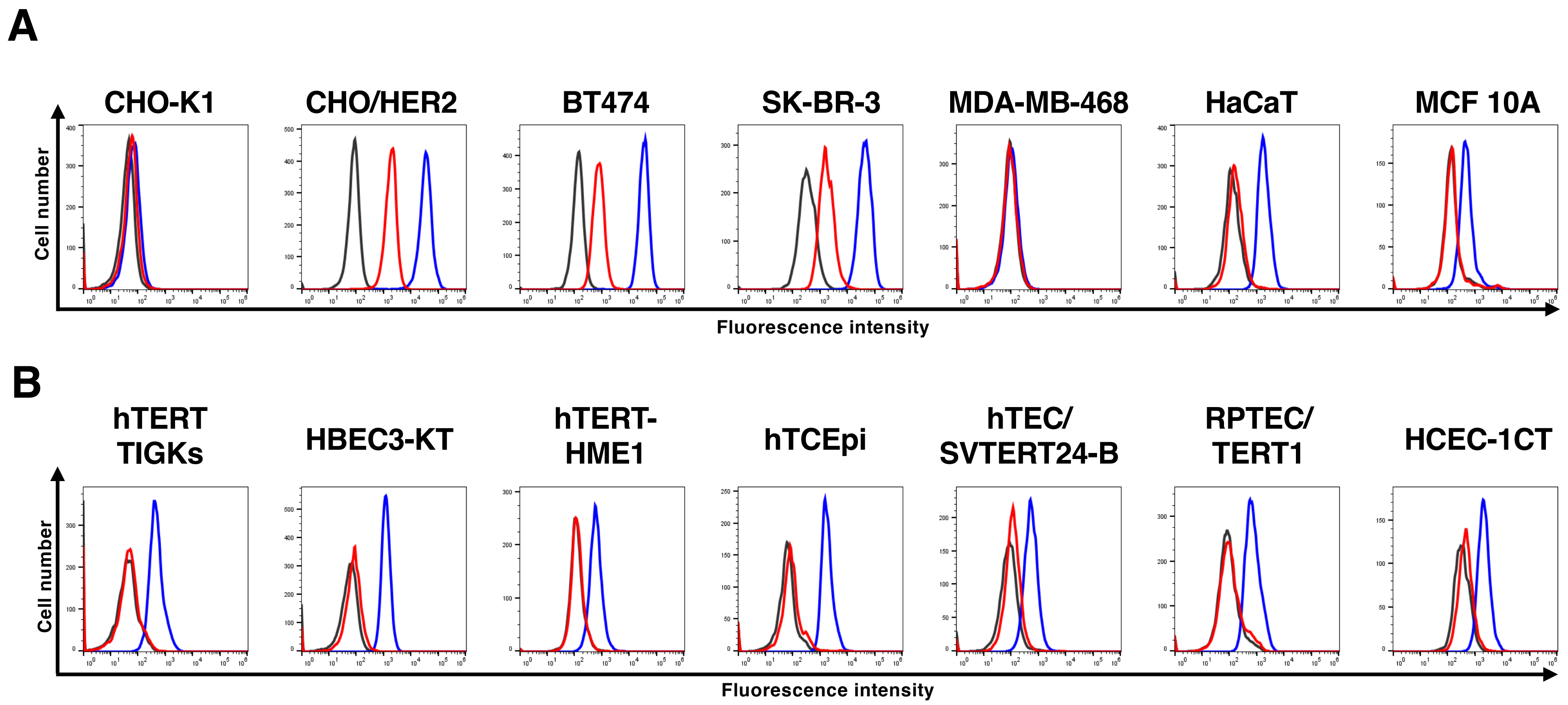

Supplementary Figure S1. Flow cytometry using anti-HER2 mAbs.
(A) Flow cytometry using H_2Mab-250-hG$_1$ (20 μg/mL; Red line) and trastuzumab (20 μg/mL; Blue line) against CHO-K1, CHO/HER2, HER2-positive breast cancers (BT-474 and SK-BR-3), a triple-negative breast cancer (MDA-MB-468), and spontaneously immortalized normal epithelial cells (HaCaT and MCF 10A). (B) Flow cytometry using H_2Mab-250-hG$_1$ (20 μg/mL; Red line) and trastuzumab (20 μg/mL; Blue line) against immortalized normal epithelial cells including hTERT TIGKs (gingiva), HBEC3-KT (lung bronchus), hTERT-HME1 (mammary gland), hTCEpi (corneal), hTEC/SVTERT24-B (thymus), RPTEC/TERT1 (kidney proximal tubule), and HCEC-1CT (colon). The black line represents the negative control (blocking buffer).

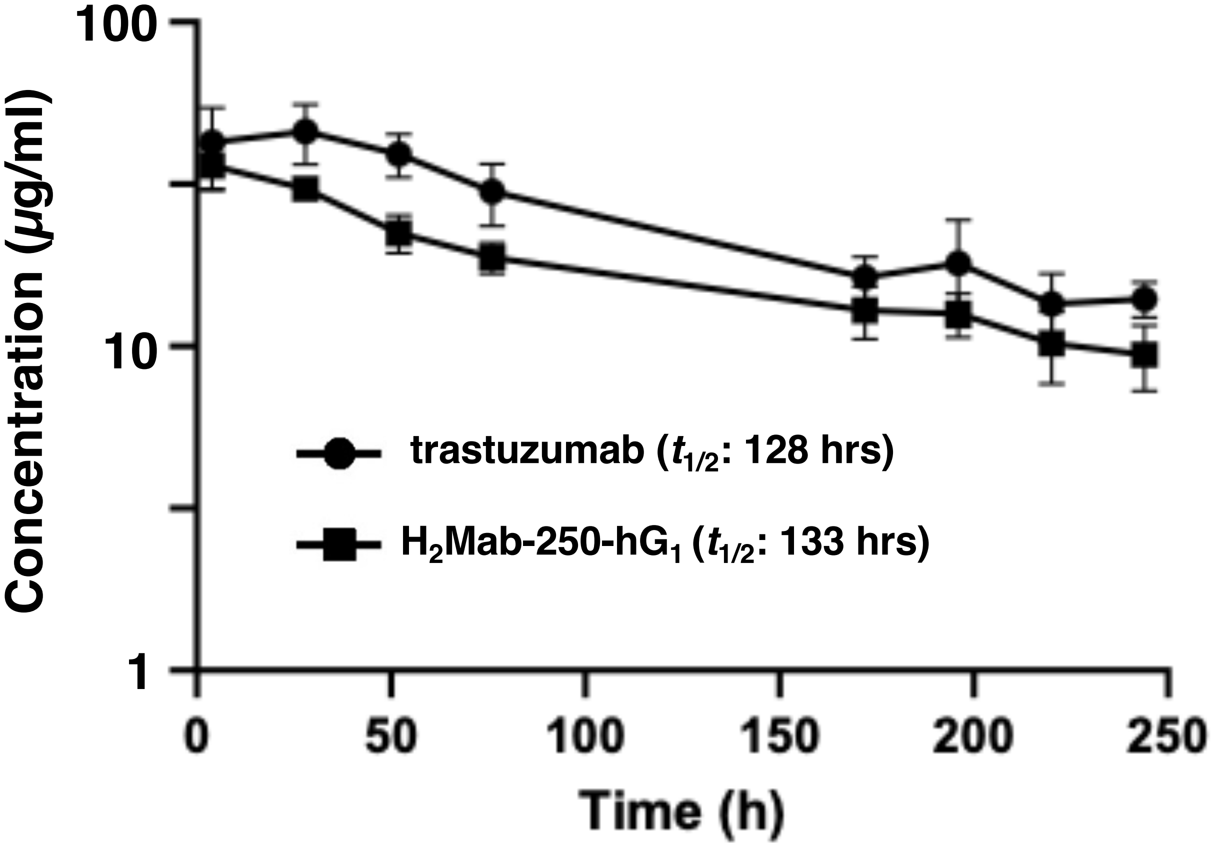

Supplementary Figure S2. The pharmacokinetics of H_2Mab-250-hG_1 and trastuzumab. H_2Mab-250-hG_1 and trastuzumab (100 μg/mouse, n=3) were intraperitoneally injected and the serums were collected from day 0 (4 hours after injection) to 10. The concentration of mAbs was determined as described in materials and methods, and the half-life ($t_{1/2}$) was estimated.

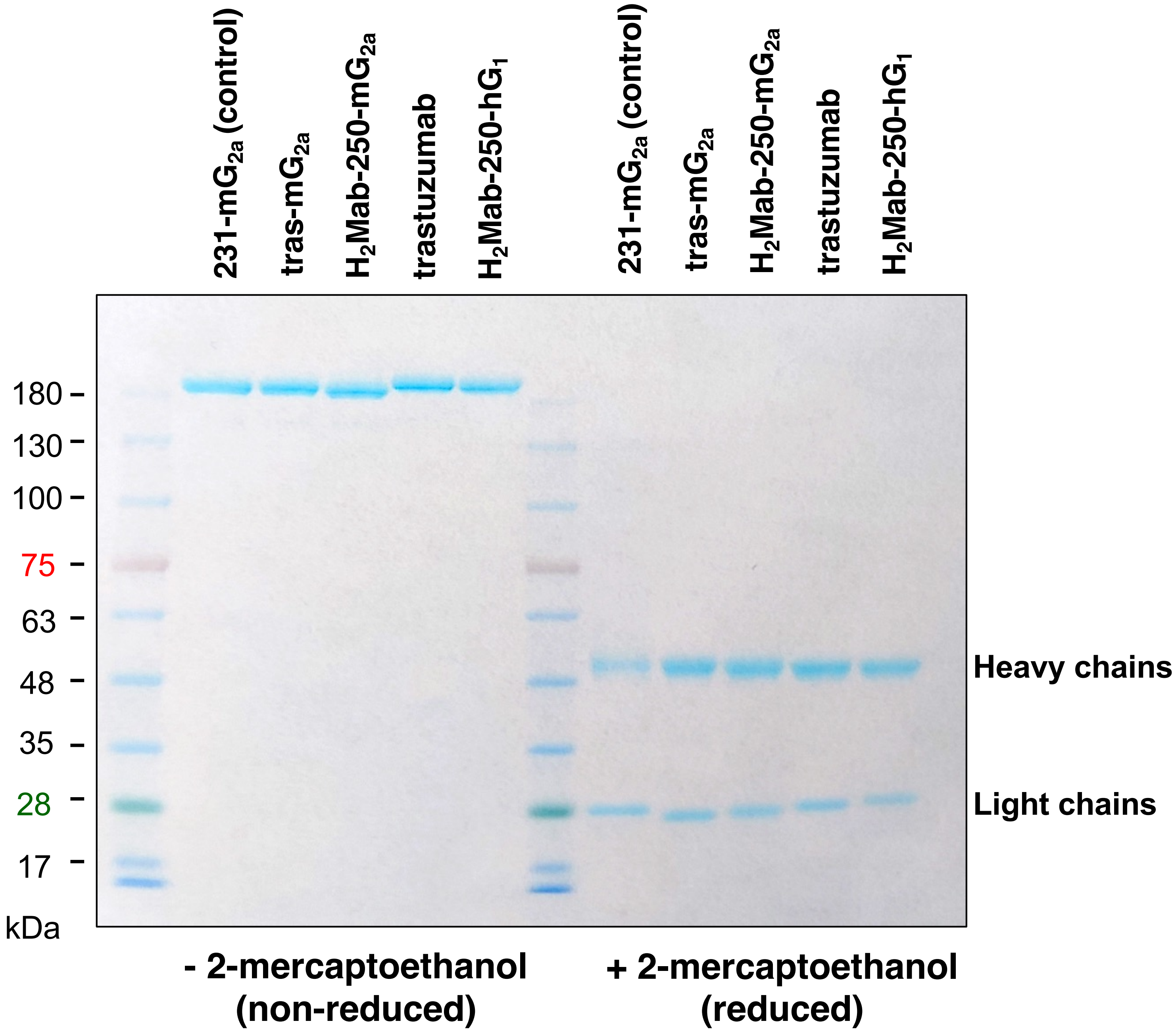

Supplementary Figure S3. Confirmation of the purified mAbs.
MAbs (2 μg) were treated with sodium dodecyl sulfate (SDS) sample buffer or SDS sample buffer without 2-mercaptoethanol (Nacalai Tesque, Inc.). Proteins were separated on 5%–20% polyacrylamide gel (FUJIFILM Wako). The gel was stained by Bio-Safe CBB G-250 Stain (Bio-Rad Laboratories, Inc.).
